# Regional medical practice variation in high-cost healthcare services: Evidence from diagnostic imaging in Austria

**DOI:** 10.1007/s10198-021-01298-w

**Published:** 2021-04-15

**Authors:** Michael Berger, Thomas Czypionka

**Affiliations:** 1 Department of Health Economics, Center for Public Health, Medical University of Vienna, Kinderspitalgasse 15/1, 1090 Vienna, Austria; 2 Institute for Advanced Studies, Josefstädterstraße 39, Vienna, Austria; 3 London School of Economics and Political Science, Houghton Street, London, WC2A 2AE UK

**Keywords:** Healthcare service utilization, Magnetic resonance imaging, Medical practice variation, Health policy, Blinder–Oaxaca decomposition, C33, H510, I18

## Abstract

Magnetic resonance imaging (MRI) is a popular yet cost-intensive diagnostic measure whose strengths compared to other medical imaging technologies have led to increased application. But the benefits of aggressive testing are doubtful. The comparatively high MRI usage in Austria in combination with substantial regional variation has hence become a concern for its policy makers. We use a set of routine healthcare data on outpatient MRI service consumption of Austrian patients between Q3-2015 and Q2-2016 on the district level to investigate the extent of medical practice variation in a two-step statistical analysis combining multivariate regression models and Blinder–Oaxaca decomposition. District-level MRI exam rates per 1.000 inhabitants range from 52.38 to 128.69. Controlling for a set of regional characteristics in a multivariate regression model, we identify payer autonomy in regulating access to MRI scans as the biggest contributor to regional variation. Nevertheless, the statistical decomposition highlights that more than 70% of the regional variation remains unexplained by differences between the observable district characteristics. In the absence of epidemiological explanations, the substantial regional medical practice variation calls the efficiency of resource deployment into question.

## Introduction

In their quest to align healthcare expenditure with actual revenues, policy makers can find viable options in the reduction of wasteful spending. The Organisation for Economic Co-operation and Development (OECD) defines wasteful spending on health as expenditures that do not yield adequate medical benefits for patients [1]. By reducing wasteful spending, policy makers can increase the efficiency of healthcare systems without cutting back on vital services. However, spotting wasteful spending in practice is not trivial. Medical practice variation can serve as helpful signpost in these endeavours. In the absence of plausible epidemiological explanations, regional differences in the use of specific healthcare services provide ample reason for researchers to question the efficiency of resource deployment.

In this paper, we focus on magnetic resonance imaging (MRI) which is a promising target because it is an increasingly popular diagnostic measure that can generate much useful information for physicians [2] yet is comparatively expensive and resource-intensive. MRI has accordingly already become the target of initiatives like *Choosing Wisley* in the United States (US) that aim at reducing unnecessary medical tests, treatments, and procedures [3]. Spending on MRI can become wasteful primarily in three scenarios. First, if the referral is vague, MRI exams may not yield the needed results and have to be repeated (e.g. MRIs with a wrong sequence, or without contrast medium when one is needed, etc.). The second scenario is when physicians use MRI in the absence of a clear indication. Even under the assumption that physicians in this case have only the patients’ best interest in mind and use MRI as a precaution' not to miss something important’, there is not much benefit to gain. The literature does not provide evidence that the substantial costs of aggressive testing strategies bear any relation to the modest yield of useful information, not least due to the high probability of incidental findings [4–6]. This brings us to the third scenario: the main problem with MRI in this respect is that it can detect all sorts of anomalies in organs that do not cause any symptoms but lead to uncertainty and thus further, often invasive, procedures. Incidental findings are hence a frequent phenomenon accompanying MRI in diagnostics, which are problematic when the diagnosis is mainly the result of a pathological finding. For instance, when MRI is used to clarify the cause of acute knee problems, and the MRI exam reveals a meniscus rupture, the rupture is often presumed to be the cause of the knee problems. However, Englund et al. [7] analyse this common inference and find that 61% of patients diagnosed with a meniscus rupture through an MRI exam did in fact not report any knee problems in the month preceding the MRI exam. It is likely that the diagnosed meniscus rupture was simply a degenerative alteration that does, a priori, not indicate need for more invasive treatment. Not only do the direct costs of MRI exams then need to be considered from this perspective, but also the indirect costs that emerge from testing without good cause (e.g. follow-up visits, etc.) [8]. Excessive testing can thereby lead to substantially increased costs while the additional benefit for patients remains doubtful.

This downside of MRI (mis-)use is widely discussed in the scientific literature: in a large systematic review, Elshaug et al. [9] classify common low-value healthcare practices, including MRI exams for lower back pain, which is also listed in the *Choosing Wisely* initiative mentioned earlier [10]. Nevertheless, even such explicitly formulated advice may fall on deaf ears. For instance, Linder et al. [11] find that in Germany clinical guidelines for the use of MRI for back pain are in many instances not adhered to. According to official guidelines, physicians are advised to treat conservatively for 6 weeks before referring a patient to an MRI exam after the initial diagnosis of acute non-specific back pain. With every third patient, physicians did not adhere to this waiting period. Also in the US, the problem of tackling unnecessary diagnostic imaging remains pressing [4]. Against this background, it is clear that the specific dynamics behind the regional variations of MRI utilization need to be evaluated to provide clear guidance for policy action when the rate of diagnostic imaging is high. The literature consists primarily of country-level analyses [12–15]. While some conclusions allow for a more universal interpretation—for instance the impact of socioeconomic status [15]—some conclusions are very specific to the country under investigation. In how far this evidence can be generalized to other countries without reference to shared healthcare system characteristics remains unclear.

The evidence from our study comes from Austria and is of interest due to Austria’s high rate of MRI exams per capita and its strong (regional) fragmentation in the public financing and governance of healthcare. Austria has one of the highest rates of MRI exams per 1000 inhabitants among OECD countries when both inpatient and outpatient setting are considered [16].^1^ Discussing the high MRI utilization in Austria, Emprechtinger et al. [16] suggest that a possible reason could lie in the use of MRI as a tool for primary diagnostics. But it is unclear whether MRI is used in this way to the same extent in all nine states. Variations could also occur at the district level, or even the physician level. An earlier study by Czypionka and Berger [18] revealed strong geographical variation in the age-standardized number of MRI exams per 1000 inhabitants on the district level in Austria which may reflect such medical practice variation and in combination with high MRI utilization rates further signal misuse of MRI. Such regional variations in the utilization of diagnostic imaging are not unique to Austria. Gransjøen et al. [12] highlight the strong regional variation in the use of diagnostic imaging of musculoskeletal diseases in Norway and find, for instance, that differences in the rate of MRI of the shoulder can be more than 100%. Explanatory factors are not investigated in this study, however. In contrast, Pransky et al. [13] investigate the inter-state differences in early MRI for acute work-related pain in the lower back in the US and find the differences largely explained by the rate of non-hospital MRI sites and the median state income.

For a better understanding, we briefly address peculiarities in the Austrian institutional framework important for the context of our analysis. Firstly, reimbursement and referral procedures differ between healthcare sectors, owing to Austria’s fragmented system of healthcare financing. Insurees receive MRIs in the outpatient sector for free at radiology institutes that are contracted by SHI upon referral, but have to pay out-of-pocket if they choose a non-contracted provider or are not referred^2^ (we will henceforth use the term *non-contract* to refer to institutes or physicians without SHI contract in this article). Secondly, the SHI funds used their limited autonomy in restricting the access to MRI services by setting requirements for referrals. Some—but not all—SHI funds in addition required that the MRI referral was approved by the head physician of the SHI fund prior to the MRI exam taking place.^3^ Thirdly, during the study period of this article, non-hospital radiology institutes were only reimbursed for their services up to a predefined annual cap on expenses (negotiated between the SHI funds and the Austrian Economic Chamber) that has in the meantime been lifted. The reimbursable expenses per service provider are capped regardless of the actual frequencies with the intention of better controlling cost growth and to reflect fixed cost degression. The cap led to substantially longer waiting periods for MRI appointments and a shift to private healthcare provision that offered quicker access to healthcare which was also encouraged by the radiology institutes [20].

Our aim in this article is to determine the extent of regional medical practice variation of high-cost diagnostic imaging in a healthcare system characterized by a social health insurance (SHI) system with comprehensive coverage and regional autonomy of payers to regulate access. This common denominator facilitates the generalization of our results among healthcare systems that share these traits. We control for regional characteristics on the district level—epidemiological factors, supply side factors, CT substitution and regulated access to imaging services—that potentially contribute to the variation in MRI utilization and decompose the regional variation into its contributing factors. We propose that the component not attributable to the observed district characteristics can be interpreted as the extent of the regional medical practice variation.

## Data and variables

We utilize a set of routine healthcare data provided by the Main Association of Austrian Social Security Institutions on the healthcare consumption of Austrian patients. The study cohort contains reimbursement data for all patients with an outpatient MRI exam between the beginning of the third quarter 2015 and the end of the second quarter 2016. No restrictions were made according to age, sex, nationality or residency. The information that patients had an MRI exam is taken from the KAL-code (Katalog Ambulanter Leistungen, catalog of ambulatory services),^4^ that specifies the services patients consumed. The dataset covers 489,190 patients with 589,801 outpatient MRI exams between the beginning of the third quarter 2015 and the end of the second quarter 2016.

For the purpose of this study, we aggregated the individual MRI episodes for the patients’ area of residence at the district level. We chose the district level as this was the smallest geographical unit for that information was available in our dataset. Moreover, healthcare services in Austria are organised in *care regions* (‘Versorgungsregionen’)^5^ that make use of the same borders. This is crucial for the linkage with epidemiological data in a later stage of the study. 2747 MRI exams were excluded from the sample as the information on the patients’ area of residence was missing, resulting in a total of 587,054 outpatient MRI exams. Healthcare services provided in outpatient departments of hospitals were provided by the Austrian Ministry of Labour, Social Affairs, Health and Consumer Protection and added to the dataset. In total, there were 172,769 MRI episodes in outpatient departments of hospitals in the study period. As this complementary data no longer included one district that was merged in 2017, this district was dropped in the later stages of the analysis, reducing the sample to 116 districts. In the complementary data, the patients of the abolished district were already allocated to the four other districts to which it was appended. The extent of this bias is unclear.

Healthcare contacts with non-contract physicians (‘Wahlärzte’) or contacts paid out-of-pocket by the patients are not included in the data. This missing information limits the explanatory power of the study design to a certain extent, as some relevant cases remain unobserved. Moreover, we do not include inpatient MRI exams, i.e. MRI exams of patients who have been admitted to a hospital, in the dependent variable as they are not substitutes for outpatient MRI exams. It is quite unlikely (though not unheard of) that patients are admitted to a hospital for the sole purpose of an MRI exam. Note that this does not rule out MRI exams in outpatient departments of hospitals, which are included in the dataset.

No information about the income or the socioeconomic status is available on the patient level. Leaning on Lambregts and van Vliet [21], we use a socioeconomic status (SES) score based on the characteristics of the patients’ area of residence. We base our composite variable on the following information on the district level: (1) the percentage of persons with only mandatory schooling in the labour force [22], (2) the percentage of unemployed persons in the labour force [22], and (3) the average net income [23]. For each variable, the districts are divided into quartiles. The higher the quartile, the worse a district is ranked in the socioeconomic dimension. The SES score is simply the average of the three quartile ranks multiplied by $-1$, allowing the measure to maintain an intuitive interpretation with lower score indicating a lower socioeconomic status.

Data on epidemiological characteristics are taken from several sources. The Austrian Health Interview Survey 2014 [24] is a representative survey that provides data on health-related behaviour on the level of the care regions. The data allow to account for differences in epidemiological factors across care regions. The rates are an approximation to the burden of drinking and smoking (due to their association with diseases that can require the use of MRI, like colorectal cancer or cadiovascular heart disease), chronic back or neck pain, the number of accidents and problems with access to care. For smoking, we use the percentage of respondents who identified as regular (daily) or occasional smokers as percentage of all respondents from the respective care region. For drinking, we use the percentage of respondents who declared to be drinking daily or almost daily in the preceding 12 months as percentage of all respondents from the respective care region. The prevalence of chronic back or neck pain is the number of respondents who reported having these types of chronic pain as a percentage of all respondents from the respective care region, and the rate of accidents is analogously derived from the number of respondents who reported having had an accident (at home, during their spare time activities or in traffic) in the preceding 12 months. The proxy for problems with access to care is derived from the number of respondents who reported they the had experienced delays in receiving treatment due to the geographical distance to the health service provider. Additional data on the mortality due to cancer on the district level are taken from official statistics [25]. Direct information on the incidence or prevalence of cancer is not available on the district level due to concerns about the data quality on this level.

As different patients have a different need for treatment, we control for the patients’ burden of disease on the district level. We use the patient-level information on all contacts with the healthcare systems for two quarters prior to the first outpatient MRI exam in the cohort-defining period and two quarters following their last outpatient MRI exam in that period to construct a proxy for the burden of disease. For this purpose, we define patients with 100 or more healthcare contacts over these two years as heavy utilizers and use the number of heavy utilizers per 1000 inhabitants in the district as a control variable in the regression.

We further control for some supply side factors that could influence the demand for healthcare services. These include the number of MRI devices in the outpatient sector, in hospitals, and devices operated in non-contract radiology institutes. The number of physicians (all specialities, including dentists) with an SHI contract and the number of non-contract physicians per district are also included. It cannot be ruled out that CT could be used as a quicker and less cost-intensive substitute for MRI, even though this practice would not be unproblematic: CT exposes patients to high doses of radiation and has different areas where application is recommended due to its completely different operating principle. We hence also include the total number of CT exams on the district level (based on the patients’ area of residency) that were performed in the study period.

We include a dummy variable accounting for the autonomy of payers to impose a restriction on the access to diagnostic imaging in the Austrian healthcare system. The majority of patients is insured with one of the state SHI funds, of which there is one for private sector employees of each state. On average, 83% of all outpatient MRI episodes in the study period within a district were claimed with a state SHI fund. Some—but not all—of these SHI funds require patients not only to have a referral by a physician, but also to have this referral approved by the SHI fund’s head physician prior to the MRI exam.^6^ Without prior approval, the costs for the MRI are not reimbursed. The idea behind this measure is to have better control over healthcare costs. This additional bureaucratic threshold could reduce the number of MRI exams if non-necessary procedures are not approved by the SHI, or when physicians in anticipation of this already change to a more conservative referral behaviour, or when patients opt for non-contract MRI institutes for these services. Therefore, we expect states without a required prior medical approval to have higher MRI examination rates. There are two limitations that have to be kept in mind for this analysis. Patients cannot choose their SHI fund freely, but are allocated according to their vocation. For some vocations (state employees, farmers, etc.), there are SHI funds that operate nationwide. Again some, but not all, of these require pre-approval for MRI exams. Additional inaccuracy occurs when patients live in a different state than they work in, as the MRI exam rates are based on the patients’ areas of residency.

Finally, we include control variables that capture demographic factors like the portion of the female population, the portion of the population aged 45–64 (the age group among which MRI exams are most common), and a dummy variable for urban regions that takes the value of 1 for state capitals and districts with $>500$ inhabitants/km$^{2}$ and 0 otherwise. The summary statistics of the variables used in the regression analysis are reported in Table 1.

**Table 1:** Summary statistics of the variables used in the regression analysis

Summary statistics					
Variable	Observations	Mean	Std. Dev.	Min	Max
**MRI exams (district level)**					
Outpatient MRI exams	117	5017.556	3597.253	110	23,913
Outpatient MRI exams per 1000 inhabitants	117	66.925	21.999	7.523	116.781
Outpatient MRI exams (including hospital outpatient departments)	116	6468.905	4050.762	115	27,066
Outpatient MRI exams per 1000 inhabitants (including hospital outpatient departments)	116	87.450	14.742	52.377	128.687
Socio-Economic Score (district level)	116	$-2.5$	$-0.790$	$-4$	$-1$
**Epidemiological factors**					
Share of smokers (care region level)	116	28.776	4.284	20.079	36.650
Share of daily drinkers (care region level)	116	5.930	1.371	3.458	8.566
Share of population with chronic back pain (care region level)	116	23.747	2.963	19.121	31.988
Share of population with chronic neck pain (care region level)	116	19.216	2.903	14.945	26.225
Share of population with accident at home (care region level)	116	2.913	0.843	1.623	4.675
Share of population with accident in spare time (care region level)	116	5.996	1.307	3.534	9.221
Cancer mortality (deaths per 1000 inhabitants; district level)	117	2.408	0.622	0.865	4.875
Share of population experiencing problems with access to care (care region level)	116	1.429	0.595	0.531	3.072
Share of heavy utilizers of patients with MRI exams (district level)	117	48.835	9.411	24.484	65.826
**Supply-side factors**					
MRI units in hospitals (district level)	117	0.786	1.401	0	9
Outpatient MRI units (district level)	117	0.547	1.030	0	8
MRI units in non-contract facilities (district level)	117	0.325	0.585	0	2
Outpatient CT examinations per 1000 inhabitants (district level)	116	35.659	16.522	0.969	103.587
CT examinations in outpatient departments in hospitals per 1000 inhabitants (district level)	116	50.506	36.368	10.466	230.813
Physicians (all types) with SHI contract per 1000 inhabitants (district level)	116	1.394	0.725	0.741	7.495
Physicans (all types) without SHI contract per 1000 inhabitants (district level)	116	1.605	2.470	0.164	22.363
Orthopaedists with SHI contract per 1000 inhabitants (district level)	116	0.045	0.060	0	0.548
Orthopaedists without SHI contract per 1000 inhabitants (district level)	116	0.168	0.225	0	1.706
Required pre-approval of MRI examinations by SHI (state level)	117	0.427	0.497	0	1
**Demographic factors (district level)**					
Share of female population	117	50.850	0.799	49.259	53.689
Share of population aged 45 to 64	117	28.932	2.133	23.031	33.223
Urbanicity (urban = 1, rural = 0)	117	0.291	0.456	0	1
Population	117	74,363	44,369.170	1911	280,258

The original sample contained information on patients in 117 districts, whereas any complementary data added at later stages of the study (e.g. MRI exams in outpatient departments in hospitals, epidemiological factors, and so forth) were only available for 116 districts. This issue arises because one district has been merged with other districts in 2017 and how patients from this district were handled differed between dates of data extraction. The assignment of the patients could not be harmonized retrospectively. We therefore report the descriptive statistics for the original sample with 117 districts, as these patients are also contained in the complementary data, but are assigned to other districts

## Methods

**Fig. 1:**
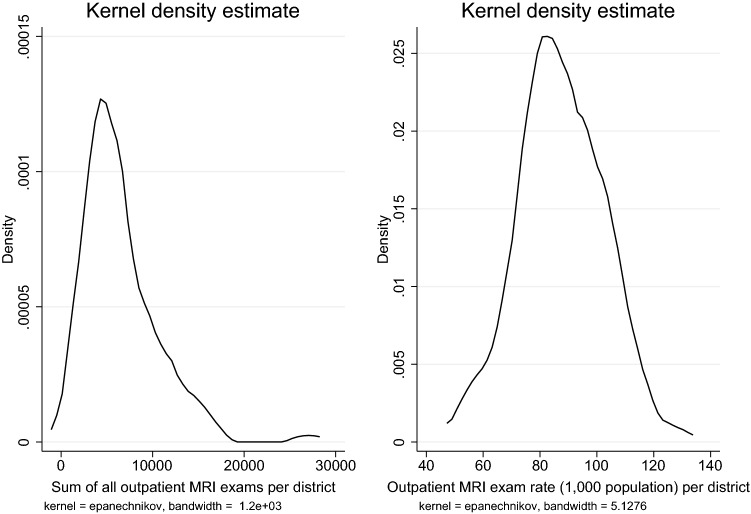
Distribution of the outcome variable as the total number of MRI exams (left) and the rate of MRI exams per 1,000 inhabitants (right) per district

Our statistical of the data consists of two steps: in the first step, we use multivariate regression models to identify the influence of the selected district characteristics. We run generalized (non)-linear regression for two dependent variables, MRI exams per 1000 inhabitants and the number of MRI exams (adding total population size as an additional control variable). While for the regression on MRI exams per 1000 inhabitants estimation by ordinary least squares is appropriate, the distribution of the number of MRI exams is non-normal and skewed to the right (Figure 1) requiring estimation by a generalized model with a negative binomial (NB) distribution for the dependent variable and the canonical log-link function. A Moran test rejected a spatial correlation structure in the error-term using a contiguity spatial weighting-matrix and a spatial weighting-matrix based on the inverse of the distance between two districts in the full model specifications at reasonable significance (*p* value $>0.1$). In the second step, we use the decomposition method developed by Blinder [26] and Oaxaca [27] (henceforth referred to as Blinder–Oaxaca decomposition) in its original form and adapted for the use with nonlinear NB regression suited for count data in Stata [28, 29]. This decomposition method is often used in labour economics to highlight unexplained wage differentials, but it has also been used to highlight differences in healthcare consumption among different patient groups [30], ethnic groups [31–33] or health status and behaviour [34–36]. The method decomposes the observed differences between two groups in the outcome variable into a part explained by observable characteristics and a part that is due to the differences in the estimated coefficients. In our study, we divide the 116 districts into a high MRI-utilization and a low MRI-utilization group (districts with MRI exams per 1000 inhabitants above and below the sample median, respectively). By applying the Blinder–Oaxaca decomposition, we highlight how much of the difference in MRI consumption between high and low MRI-utilization districts is explained by observable characteristics and how much is owed to unobserved differences, which can be interpreted as an approximation of the difference due to medical practice variation.

### Decomposition method

The starting point of our decomposition approach is our basic linear multivariate regression model given by:

1 $$\begin{aligned} Y_{i} = X_{i} \beta _{i} + \varepsilon _{i} \end{aligned}$$

where $Y_{i}$ is the dependent variable of the model (here the rate of MRI exams per 1000 inhabitants), $X_{i}$ is the vector of the control variables and $\varepsilon _{i}$ is the error term for the districts $i=1,\dots ,N$.

In the basic form of the Blinder–Oaxaca decomposition, the linear regression model (1) is estimated separately for two groups $g=1,2$

2 $$\begin{aligned} Y_{g} = X_{g} \beta _{g} + \varepsilon _{g} \end{aligned}$$

Under the assumption of a linear model, Oaxaca and Ransom [37] express a generalized form of the gap in the mean outcome for the two groups by

3 $$\begin{aligned} \bar{Y}_{1} - \bar{Y}_{2} = ( \bar{X}_{1} - \bar{X}_{2} ) \beta ^{*} + \bar{X}_{1} (\beta _{1} - \beta ^{*}) \bar{X}_{2} (\beta ^{*} - \beta _{2}) \end{aligned}$$

with

4 $$\begin{aligned} \beta ^{*} = \Omega \beta _{1} + (I - \Omega ) \beta _{2} \end{aligned}$$

where $\bar{Y}_{g} = {\frac{1}{N_{g}}} \sum _{i=1}^{N_{g}} Y_{gi}$ and $\bar{X}_{g} = {\frac{1}{N_{g}}} \sum _{i=1}^{N_{g}} X_{gi}$ are the group’s respective sample means, and $\Omega$ is a weighting matrix, *I* is the identity matrix and $\beta ^{*}$ is a weighted average of the coefficient vectors. While the first term on the right-hand side describes the part of the difference in the outcome between the two groups that stems from the differences in the control variables (i.e. the observable characteristics), the second term describes how much of the difference is caused by differences in the estimated coefficients ${\hat{\beta }}_{g}$. For the purpose of this analysis, we specify $\Omega = I$, thereby setting the coefficients of the low MRI-utilization group as the reference.

This decomposition requires adaption for non-linear NB-regression models (which we use with the number of MRI exams as dependent variable) as the conditional expectations $E( Y_{{\text{ gi }}}|X_{{\text{ gi }}})$ differ from ${\bar{X}_{g}} {\hat{\beta }_{g}}$. Bauer and Sinning [28] suggest an alternative decomposition of the mean difference of $Y_{g}$ using the conditional expectations evaluated at different coefficient estimates with $g=1$ as the reference group:

5 $$\begin{aligned} \Delta _{1,{\text{ NL }}}= & {} \left[ E_{\beta _{1}} (Y_{1i} | X_{1i}) - E_{\beta _{2}} (Y_{2i} | X_{2i}) \right] \nonumber \\&\quad + \left[ E_{\beta _{1}} (Y_{2i} | X_{2i}) - E_{\beta _{2}} (Y_{2i} | X_{2i}) \right] \end{aligned}$$

where $E_{\beta _{g}}(Y_{gi} | X_{gi})$ is the conditional expectation of $Y_{gi}$ and $E_{\beta _{g}}(Y_{hi} | X_{hi})$ is the conditional expectation of $Y_{hi}$ evaluated at $\beta _{g}$ with $g,h = 1,2$ and $g \ne h$. Again, the first term on the right-hand side describes the part of the difference in the outcome variable due to the differences in the control variables. The decomposition is applied to NB-regression models by replacing the conditional expectation $E_{\beta _{g}} (Y_{gi}|X_{gi})$ with the respective sample counterpart of the conditional mean of the NB-regression model given by [28]:

6 $$\begin{aligned} S ( \hat{\beta }_{g, {\text{ NB }}}, X_{gi}) = \bar{Y}_{g, \hat{\beta }_{g, {\text{ NB }}}} = \frac{1}{N} \sum _{i=1}^{N_{g}}{\text{ exp }} \left( X_{gi} \hat{\beta }_{g, NB} \right) \end{aligned}$$

## Results

**Fig. 2:**
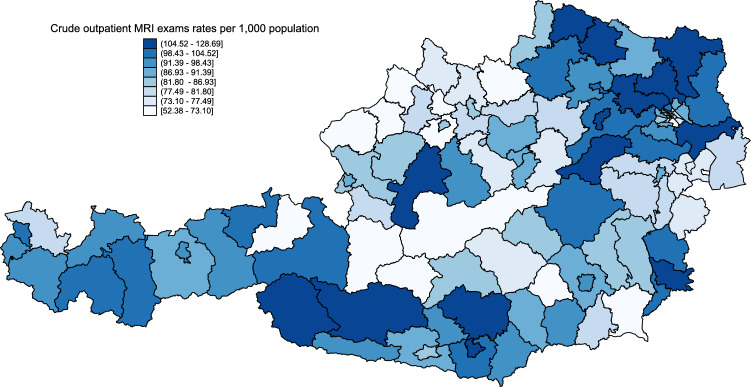
Crude utilization rates of outpatient MRI exams per 1000 inhabitants in Austria from Q3-2015 to Q2-2016

The data reveal substantial geographical variation in number of MRI exams per 1000 inhabitants on the district level in Austria ranging from 52.38 to 128.69 (Fig. 2). While the districts with the highest rates of exams per 1000 inhabitants in the outpatient sector are located in Tyrol and Lower Austria, some districts in Upper Austria and Styria have very low MRI utilization with rates not even half of those found in the high MRI-utilization districts. There is no clear geographical pattern in MRI utilization, although they seem to be generally higher in the north-east and lower in the north-west. Apart from a pattern across states, there seems to be within-state variation on the district level as well. Again, no distinct pattern is observed.

### Regression results

We sequentially test four model specifications corresponding to our four categories of regional characteristics (columns M1–M4) and a combined model using OLS (MRI rate per 1000 inhabitants) and NB regression (number of MRI exams) depending on the requirements of the type of the underlying data. The results of the statistical regression analysis are provided in Table 2. At at first glance, the results strongly depend on the outcome variable of the regression. For example, the number of outpatient MRI units has a strong impact on the MRI exams per 1000 inhabitants whereas it does not have a statistically significant impact when the absolute number of MRI exams is used as the outcome. Conversely, the portion of the female population and the SES score are associated with a higher utilization when the absolute number of MRI exams is considered, but not when MRI exams per 1000 inhabitants are investigated. Only the effect of the approval requirement of the state SHI funds is statistically significant with both outcomes. The coefficients of the two estimation methods have to be interpreted differently: while the coefficients of the OLS regression are absolute changes in the outcome variable, the coefficients of the NB regression with the canonical log-link function are changes in percent.

First, we turn to the OLS results in more detail: among the base control variables, the number of outpatient MRI units has a highly significant effect (approx. $+4$ MRI exams per 1000 inhabitants for each additional unit) that is robust throughout all model specifications. Other characteristics are only statistically significant in one or two models each. However, the effect of a higher percentage of persons aged 45–64 in the population (approx. $+3$ MRI exams per 1000 inhabitants for a one percentage point increase) is arguably more robust, as the sign does not change between models, but it is not clear in which direction the causality is pointing. In contrast, the portion of heavy utilizers in the outpatient MRI patients (the proxy for the burden of disease) changes sign when the pre-approval dummy is included. Among the epidemiological factors, a higher proxy for chronic neck pain are associated with more ($+2$) MRI exams per 1000 inhabitants, whereas a higher prevalence of chronic back pain is found to have an unexpected decreasing effect ($-1.5$). On the supply side, an additional non-contract physician per 1000 inhabitants decreases the number of MRI exams per 1000 inhabitants by roughly five cases. In contrast, the number of non-contract orthopaedists per 1000 inhabitants is found to have a very large effect, each additional non-contract orthopaedist increasing the number of MRI exams per 1000 inhabitants by 36–43 exams. The size of the effect can be traced back to the rather low number of orthopaedists without SHI contract per district (see Table 1), likely giving the estimated coefficient an upward bias. Finally, while there is no indication for a possible substitution of MRI with CT, the approval requirement of state SHI funds results in a substantially reduced number of MRI exams per 1000 inhabitants (approx. $-18$).

The picture changes when we turn to the nonlinear regression results on the absolute number of MRI exams: a one percentage point increase in the portion of the female population increases MRI exams by roughly 0.1specifications). Unsurprisingly, there is a statistically significant correlation between the population size and the number of MRI exams, with 1000 additional citizens increasing the number of MRI exams by 0.01%. In contrast to the OLS results, a higher SES score is associated with a slight increase in the number of MRI exams ($+0.1$% per one unit increase). Problems in accessibility to healthcare is statistically significant only in one model specification, though both direction and magnitude are by and large consistent. Among epidemiological factors, regular drinking has a small increasing impact on MRI exams. While for the supply side factors we do not find a statically significant association, we find a minor positive correlation with the number of CT exams per 1000 inhabitants. This indicates that MRI is not substituted with CT. The approval requirement by state SHI funds again has a statistically significant and substantial effect, decreasing MRI exams by 23–24%.

**Table 2:** OLS and NB regression results of the determinants of the number of MRI exams in Austria per district

Estimation	OLS						Negative binomial regression				
Dependent variable	MRI exams per 1000 inhabitants						MRI exams				
Hypothesis	(M1)	(M2)	(M3)	(M4)	(M1)–(M4)		(M1)	(M2)	(M3)	(M4)	(M1)–(M4)
% Female	0.615	0.544	0.736	0.392	0.389		0.0995**	0.130***	0.133***	0.129***	0.0907*
	(1.126)	(0.529)	(0.593)	(0.531)	(0.739)		(0.0373)	(0.0241)	(0.0198)	(0.0199)	(0.0398)
Population (in thousands)							0.0120***	0.0127***	0.0130***	0.0135***	0.0117***
							(0.000938)	(0.00127)	(0.000978)	(0.00106)	(0.00104)
% Population 45-64	1.182	1.436	1.075	2.775**	2.822**		0.0716	0.0365	0.0234	0.0457	0.100
	(1.444)	(0.874)	(0.955)	(0.852)	(0.944)		(0.0588)	(0.0367)	(0.0316)	(0.0345)	(0.0652)
Outpatient MRI units	3.102**	3.195**	3.350**	4.317***	3.919***		$-$ 0.0366	$-$ 0.0565	$-$ 0.0549	$-$ 0.0489	$-$ 0.0203
	(1.195)	(0.979)	(1.201)	(0.935)	(0.934)		(0.0413)	(0.0495)	(0.0487)	(0.0472)	(0.0384)
Inpatient MRI units	0.511	0.350	0.232	1.501	1.371		0.0274	$-$ 0.00477	$-$ 0.00749	0.00473	0.0370
	(1.083)	(0.989)	(0.890)	(0.839)	(0.830)		(0.0374)	(0.0275)	(0.0257)	(0.0258)	(0.0368)
Non-contract MRI units	0.695	$-$ 0.659	0.748	$-$ 1.297	$-$ 1.213		$-$ 0.00823	$-$ 0.00404	0.00745	$-$ 0.0184	$-$ 0.00761
	(2.400)	(2.505)	(2.440)	(2.290)	(2.068)		(0.0490)	(0.0455)	(0.0451)	(0.0441)	(0.0513)
% Heavy Utilisers	0.179	0.296*	0.383**	$-$ 0.0676	$-$ 0.367		0.00415	0.000473	0.00138	$-$ 0.00488	$-$ 0.00182
	(0.193)	(0.139)	(0.143)	(0.169)	(0.190)		(0.00340)	(0.00287)	(0.00253)	(0.00383)	(0.00414)
Socioeconomic Status Score	$-$ 0.548	$-$ 0.965	0.602	0.314	$-$ 0.581		$-$ 0.123**	$-$ 0.135	$-$ 0.102*	$-$ 0.109**	$-$ 0.120*
	(1.662)	(1.885)	(1.620)	(1.360)	(1.706)		(0.0460)	(0.0707)	(0.0429)	(0.0423)	(0.0593)
City	$-$ 5.640	$-$ 6.336	$-$ 3.068	$-$ 3.559	$-$ 6.348		$-$ 0.204	$-$ 0.109	$-$ 0.146	$-$ 0.153	$-$ 0.119
	(4.668)	(4.709)	(4.655)	(3.893)	(4.519)		(0.146)	(0.144)	(0.157)	(0.149)	(0.143)
Accessability problems	$-$ 4.172	$-$ 4.179	$-$ 3.309	$-$ 2.300	$-$ 2.383		$-$ 0.128	$-$ 0.107*	$-$ 0.0866	$-$ 0.0757	$-$ 0.124
	(2.594)	(2.503)	(2.526)	(2.164)	(2.085)		(0.0802)	(0.0538)	(0.0529)	(0.0476)	(0.0706)
**Epidemiological factors**											
Smoking	0.213				0.186		0.00934				0.0151
	(0.528)				(0.432)		(0.0140)				(0.0133)
Regular drinking	1.322				0.631		0.0765**				0.0628**
	(1.131)				(0.905)		(0.0255)				(0.0198)
Home accidents	$-$ 0.854				$-$ 1.762		$-$ 0.0478				$-$ 0.0508
	(1.653)				(1.402)		(0.0297)				(0.0296)
Sparetime accidents	$-$ 0.375				$-$ 0.402		$-$ 0.000966				$-$ 0.00506
	(1.119)				(0.979)		(0.0217)				(0.0200)
Chronic back pain	$-$ 0.746				$-$ 1.599*		0.0196				0.0150
	(0.935)				(0.779)		(0.0170)				(0.0159)
Chronic neck pain	1.068				1.994*		$-$ 0.0251				$-$ 0.0201
	(1.022)				(0.823)		(0.0191)				(0.0169)
Cancer mortality	2.150				$-$ 0.0604		$-$ 0.0929				$-$ 0.129
	(2.447)				(1.404)		(0.0817)				(0.0954)
**Supply side factors**											
Physicians with SHI contract		4.373			7.514*			$-$ 0.171			$-$ 0.0811
		(4.952)			(3.439)			(0.138)			(0.126)
Physicians without SHI contract		$-$ 4.110			$-$ 4.849*			$-$ 0.0132			$-$ 0.0440
		(2.103)			(2.007)			(0.0545)			(0.0698)
Orthopaedists with SHI contract		15.18			1.216			0.148			0.165
		(38.22)			(29.59)			(1.169)			(1.229)
Orthopaedists without SHI contract		43.14**			36.18*			0.627			0.509
		(16.12)			(14.33)			(0.509)			(0.562)
CT exams per 1,000 inhabitants			0.0594		0.0264				0.00112		0.00131
			(0.0375)		(0.0418)				(0.000587)		(0.000693)
SHI Approval (state SHI funds)				$-$ 17.37***	$-$ 18.32***					$-$ 0.221**	$-$ 0.226**
				(3.125)	(3.120)					(0.0817)	(0.0847)
N	116	116	116	116	116		116	116	116	116	116
Log pseudolikelihood	$-$ 463.375	$-$ 461.637	$-$ 464.574	$-$ 447.948	$-$ 438.343		$-$ 1115.746	$-$ 1116.207	$-$ 1116.366	$-$ 1116.030	$-$ 1115.084
AIC	8.265085	8.183398	8.182318	7.895653	7.93695		19.5301	19.48633	19.43735	19.43156	19.62214

Robust standard errors in parentheses

*$$p < 0.05$$, **$$p < 0.01$$, ***$$p < 0.001$$

### Decomposition results

The results of the decomposition analysis for the linear and the nonlinear models are reported in Table 3. Overall, the results suggest that the regional variation in MRI utilization is largely owing to differences in the estimated coefficients and hence on the unobserved district-specific differences in MRI utilization. The estimated difference between the two MRI utilization groups is 23.6 MRI exams per 1000 inhabitants, or roughly 2000 MRI exams.

The predictions based on MRI exams per 1000 inhabitants as the outcome measure are more stable and reflect the findings of the multivariate regression analysis. Especially the required approval of state SHI funds accounts for a sizable portion of the observed regional variation. The impact is less clear when the crude number of MRI exams is used as the outcome. While in the linear decomposition both the explained (i.e. due to differences in the observable characteristics) and unexplained (i.e. due to differences in the estimated coefficients) component of the decomposition Eq. 3 are statistically significant, this is not the case for the nonlinear decomposition for the outcome based on count data and bootstrapped standard errors described in (5). It is interesting to note that the gap between the explained and unexplained component is much wider for certain model specifications (columns M2, M3 and M4).

**Table 3:** Blinder–Oaxaca decomposition results of the number of MRI exams in Austria per district

	Linear Blinder–Oaxaca decomposition									
	Dependent variable = MRI exams per 1000 inhabitants									
	(M1)		(M2)		(M3)		(M4)		(M1)–(M4)	
	Coefficient	in % of $\Delta$	Coefficient	in % of $\Delta$	Coefficient	in % of $\Delta$	Coefficient	in % of $\Delta$	Coefficient	in % of $\Delta$
Raw difference $\Delta$	$-$ 23.62***		$-$ 23.62***		$-$ 23.63***		$-$ 23.63***		$-$ 23.61***	
	(1.821)		(1.791)		(1.753)		(1.750)		(1.885)	
Explained	$-$ 2.041	8.6	$-$ 1.972	8.3	$-$ 1.946	8.2	$-$ 4.845	20.5	$-$ 6.208	26.3
	(1.920)		(2.085)		(1.688)		(2.486)		(3.266)	
Unexplained	$-$ 21.58***	91.4	$-$ 21.65***	91.7	$-$ 21.68***	91.8	$-$ 18.79***	79.5	$-$ 17.40***	73.7
	(2.626)		(2.694)		(2.277)		(2.669)		(3.904)	

	Nonlinear (negative binomial) Blinder–Oaxaca decomposition									
	Dependent Variable = MRI exams									
	(M1)		(M2)		(M3)		(M4)		(M1)–(M4)	
	Coefficient	in % of $\Delta$	Coefficient	in % of $\Delta$	Coefficient	in % of $\Delta$	Coefficient	in % of $\Delta$	Coefficient	in % of $\Delta$
Raw difference $\Delta$	2031.927**		2035.388**		2102.078**		2078.747**		1947.811*	
	(698.855)		(727.439)		(721.557)		(765.450)		(781.497)	
Explained	488.383	24.0	953.907	46.9	960.248	45,7	994.762	47.9	584.719	30.0
	(1830.474)		(1146.082)		(1091.650)		(1383.137)		(2340.863)	
Unexplained	1543.543	76.0	1081.482	53.1	1141.83	54,3	1089.958	52.1	1363.092	70.0
	(1409.706)		(711.346)		(608.519)		(907.727)		(2058.583)	

(Robust) standard errors in parentheses; Bootstrapped standard errors in nonlinear decomposition based on 100 replications

The reference group are districts with outpatient MRI utilization below the sample median

*$$p \le 0.05$$, **$$p \le 0.01$$, ***$$p \le 0.001$$

## Discussion

Our statistical analysis suggests that regional variation in MRI utilization in Austria is not rooted in epidemiology. The dynamic behind the regional variation of MRI utilization thus differs from that of healthcare services in general which, in recent empirical work, is by and large explained by differences in patient characteristics [38, 39]. It has been proposed that the reasons for regional variation differ between institutional settings and types of care [40], but they may even vary across the spectrum of healthcare services.

Supply side factors, too, do not provide a satisfactory answer to the causes of the regional variation in MRI utilization. Although one may interpret our results as an indication that the supply side trumps the demand side, it still fails to account for the magnitude of the regional variation in MRI utilization. We find a positive association between the number of MRI exams per 1000 inhabitants and the number of MRI units, but the direction of the effect is not a priori clear. Indeed, the number of operating MRI units is higher than foreseen on the national level by policy makers in the Austrian structural plan for large medical equipment (*Österreichischer Strukturplan Gesundheit–Großgeräteplan* [41]). It is not unreasonable to assume that MRI providers will strive to operate their machinery at a capacity compatible with running a profit, conceivably by making use of their personal networks to influence referral behaviour. In the context of the Austrian healthcare system, such ‘old boys’ networks’ indeed seem to impact the referral behaviour of physicians [42]. Our finding that a higher demand for MRI scans is not warranted by patients’ need for treatment in terms of epidemiological factors further hints at the channel of supplier-induced demand. The results concerning the influence of the private healthcare sector remain ambiguous due to the lack of data on non-contract healthcare contacts. While the negative association between the number of non-contract physicians and the number of MRI exams per 1,000 inhabitants may be read as a indication of a general shift towards private care, we caution to assume that patients frequenting non-contract physicians will choose to get MRI exams at non-contract facilities as well, as an MRI costs several hundred euros. In fact, previous research from Austria suggests lively movement of patients between contract and non-contract care providers [43]. This is also reflected in the the strong positive association between non-contract orthopaedists and the number of MRI exams per 1000 inhabitants contrasting the negative association of non-contract physicians in general. On a positive note, the results do not suggest that physicians substitute MRI with CT exams, which are less cost-intensive but expose patients to higher radiation doses and may not be the correct tool from a diagnostic perspective. The two diagnostic procedures, in fact, seem to move in tandem, though the association is small and not statistically significant in our data. The minor positive association could be a residue of instances when a new large radiology center or department offering both CT and MRI is opened. Capacities for both services are thereby expanded leading to a simultaneous, though otherwise unrelated, positive association.

A crucial finding of our analysis is that district MRI rates are considerably lower when state SHI funds use their ability to restrict access to MRI services. The regional variation in MRI utilization is, therefore, also a direct consequence of the payers’ regulatory autonomy in the Austrian healthcare system. This result is potentially relevant also in the context of other SHI-financed healthcare systems. Although our analysis can only focus on the guidelines and requirement of the state SHI funds (thereby excluding nationwide SHI funds that do and do not require pre-approval of MRI exams), the effect is still quite substantial. Whether the head physicians at the SHI regularly deny MRI exams that are considered unnecessary, or if the behavioural changes already take effect at the level of the treating physicians or patients (who may be deterred by the bureaucratic threshold), remains unclear. Moreover, as the dataset does not cover MRI exams in private clinics that are paid out-of-pocket by the patients, it cannot be ruled out with certainty that patients simply shift their healthcare consumption to the private sector, although this effect would be limited by the fact that they would have to pay several hundred euros out-of-pocket. In the absence of the necessary country-level data, future research could aim to synthesize evidence from other countries with comparable healthcare systems characteristics to answer these open questions.

### Limitations

Our analysis faces some limitations that are crucial for the interpretation of our results. Firstly, we cannot rule out the possibility that the observed regional differences are exacerbated by data issues as epidemiological data are not available on the district level and data on healthcare service consumption in non-contract practices were not available at all. However, we think that is unlikely that differences in the uptake of MRI exams in non-contract facilities are the main culprit behind the observed regional disparities in MRI exam rates given their smaller market share compared to public providers. We consider it more likely that these are caused by the differences in referral behaviour of physicians e.g. in the presence of ‘old boys’ networks’ [42]. Secondly, we stress that our study design does not allow for a causal interpretation of the results. Our results are first and foremost descriptive and explorative. The true extent of the regional differences cannot be captured in this analysis, though we are confident that our estimate is a suitable first assessment of the situation. In-depth consultations with local experts, case studies, and analyses of physicians’ referral behaviour are directions for future research that can yield pivotal information to support reform processes.

## Conclusion

MRI use is comparatively high in Austria and previous research has shown that there is substantial regional variation. Our study provides a first exploratory investigation into the causes of the regional variation in MRI utilization in Austria. As MRI is a high-cost procedure, its use should be indicated by the patients’ need for treatment only. However, the empirical evidence of our present study points towards medical practice variation that is not rooted in regional epidemiology. In the absence of a plausible epidemiological explanation, the substantial regional medical practice variation is a strong indicator for inefficiencies in the utilization of healthcare resources.

The sheer magnitude is remarkable. More than 70% of the regional variation in MRI utilization in Austria remains unexplained in our statistical analysis. As a consequence, there is plenty of potential in streamlining the utilization of MRI, for instance by fostering the use of nationwide clinical guidelines or improved, clear communication between referring physician and radiologist (e.g. by electronic referral) as well as between patient and physician about the correct indication for MRI. Though our results further suggest that regional policy can find some leverage in controlling MRI utilization in their autonomy in restricting access to diagnostic imaging services, harmonized nationwide action would be preferable to avoid further exacerbating regional variation in utilization.

## Footnotes

1 The official OECD statistics are misleading as in contrast to several other countries, only inpatient services are reported for Austria [17]. However, the majority of MRI exams in Austria occurs in an outpatient setting. When all sectors are considered in the dataset of this analysis, the overall number of MRI exams per 1000 inhabitants is 115.4 in the period from Q3-2015 to Q2-2016.

2 Patients can file for a refund of up to 80% of the official fee in the respective tariff catalogue, although privately paid tariffs are usually considerably higher than what is reimbursed by the SHI institutions [19].

3 In the beginning of 2020 the state SHI funds were merged and a gradual harmonization of the services was started. One of the first steps was to lift the pre-approval requirement. As of writing this article, it is still unclear how this will affect MRI utilization.

4 As the KAL-system was introduced in 2015 we cannot rule out that some variation in the data is caused by incomplete data collection in the early roll-out phase.

5 The care regions are administrative units that span one or more districts within a state. There were 32 such care regions in 2015/2016.

6 In the state SHI funds of Burgenland and Styria, the pre-approval was only required for referrals by general practitioners (GP), but not from specialists (as long as the referral was due to an illness within their speciality). In the dichotomous variable capturing these reforms we considered the two states to have pre-approval, because empirical evidence suggests that most referrals come from GPs [18]. Including these two states in the other group only had a minor effect on the results.
